# Comparative Analysis of the Intermolt and Postmolt Hepatopancreas Transcriptomes Provides Insight into the Mechanisms of *Procambarus clarkii* Molting Process

**DOI:** 10.3390/life11060480

**Published:** 2021-05-25

**Authors:** Shengyan Su, Brian Pelekelo Munganga, Can Tian, Jianlin Li, Fan Yu, Hongxia Li, Meiyao Wang, Xinjin He, Yongkai Tang

**Affiliations:** 1 Key Laboratory of Genetic Breeding and Aquaculture Biology of Freshwater Fishes, Ministry of Agriculture, Freshwater Fisheries Research Center, Chinese Academy of Fishery Sciences, Wuxi 214081, China; susy@ffrc.cn; 2 Wuxi Fisheries College, Nanjing Agricultural University, Wuxi 214081, China; brijelimunga@gmail.com (B.P.M.); 18838256118@163.com (C.T.); lijl@ffrc.cn (J.L.); yuf@ffrc.cn (F.Y.); lihongxia@ffrc.cn (H.L.); wangmy@ffrc.cn (M.W.); hengde@aliyun.com (X.H.)

**Keywords:** intermolt, postmolt, transcriptomes, molecular mechanisms, red swamp crayfish, *Procambarus clarkii*, molting

## Abstract

In the present study, we used RNA-Seq to investigate the expression changes in the transcriptomes of two molting stages (postmolt (M) and intermolt (NM)) of the red swamp crayfish and identified differentially expressed genes. The transcriptomes of the two molting stages were de novo assembled into 139,100 unigenes with a mean length of 675.59 bp. The results were searched against the NCBI, NR, KEGG, Swissprot, and KOG databases, to annotate gene descriptions, associate them with gene ontology terms, and assign them to pathways. Furthermore, using the DESeq R package, differentially expressed genes were evaluated. The analysis revealed that 2347 genes were significantly (*p* > 0.05) differentially expressed in the two molting stages. Several genes and other factors involved in several molecular events critical for the molting process, such as energy requirements, hormonal regulation, immune response, and exoskeleton formation were identified and evaluated by correlation and KEGG analysis. The expression profiles of transcripts detected via RNA-Seq were validated by real-time PCR assay of eight genes. The information presented here provides a transient view of the hepatopancreas transcripts available in the postmolt and intermolt stage of crayfish, hormonal regulation, immune response, and skeletal-related activities during the postmolt stage and the intermolt stage.

## 1. Introduction

Crustacean rearing has become an important sector of aquaculture [1,2]. Crustaceans are rich in protein and are an excellent source of minerals and vitamins (zinc, iron and Vitamin B-12, choline, etc.), compared to finfish [2,3]. Given the rapid rate of human population growth around the world, crustacean’s contribution to meeting human protein needs will continue to rise. However, the crustacean industry has its bottlenecks, which are disease outbreaks, cannibalistic behavior, in addition to the lack of ways to enhance their growth [4]. Crustaceans undergo gradual growth, which regularly requires the shedding of their exoskeleton, known as molting, to grow in size [5]. In many cases, molting is also necessary for copulation and successful reproduction in other crustaceans [6]. This characteristic has significant implications for cannibalistic timing because after molting, the animals are unable to defend themselves and are hence highly vulnerable to cannibalism until their new shell is fully calcified. Thus far, the application of molecular techniques has solved several problems related to fisheries management, conservation, and aquaculture [7,8]; therefore, studying the molecular molting mechanisms is imperative.

Red swamp crayfish (*Procambarus clarkii)* is an important kind of crustacean, and molting is a critical process in red swamp crayfish. Similar to that of other crustaceans, the red swamp crayfish molting process is divided into four hormone-controlled continuous phases—the intermolt, premolt, molt (also known as ecdysis), and postmolt [5,9,10]. Molting is a multifaceted process controlled by elaborate regulatory factors, including neuropeptide hormones and ecdysteroids [11,12,13]. These hormones are secreted by two endocrine glands—the paired Y-organs (glands in the maxillary somites) and neurosecretory cells (medulla terminalis X-organ sinus gland (XO–SG) complex) located within the eyestalks [14,15]. The neurosecretory cell secretes neuropeptide hormone, a molt-inhibiting hormone (MIH) that prevents molting during the intermolt and postmolt stage [5,16]. In contrast, the Y-organs secrete ecdysteroids hormone (a derivative of ecdysone, 25-deoxyecdysone, and 3-dehydroecdysone) that stimulate molting. MIH circulation inhibits the synthesis of ecdysteroid by the Y-organs for most of the molt cycle (intermolt). Under favorable external and/or internal conditions (e.g., temperature, light, loss of limbs) negative feedback is sent to the XO–SG that results in decreased circulation of MIH. Reduced MIH level stimulates synthesis and release of ecdysteroid by the Y-organ, thereby initiating the premolt stage [5,17]. MIHs are involved in inhibiting the synthesis and release of ecdysteroids, and they have also been observed to play a role in reproduction [18,19].

Earlier studies have led to a deeper understanding of the physiological and endocrine processes that take place during molting [5,14,20,21], thereby giving a chance for manipulation and control of the molting processes. Thus far, molting in crustaceans can be induced by hormones or by eyestalk ablation. Eyestalk ablation shuts down all the hormones that inhibit the animal to undergo ecdysis [21,22,23], while hormonal control (melatonin, α-ecdysone, etc.) suppresses the production of MIH, which, in turn, initiates the production of ecdysteroids, thereby leading to molting [24]. Although the physiological and endocrinal mechanisms of the molting process have been widely studied, a lot regarding molecular mechanisms of molting in crustaceans is yet to be revealed and learned. Moreover, studies on crayfish molecular molting mechanisms are still few; hence, these mechanisms remain poorly understood.

To the best knowledge of the authors, only the expression of cytoskeletal and molt-related genes has been conducted to elucidate the molecular mechanisms underlying the red swamp crayfish molting process [25]. In that study, Tom et al. identified several genes and other factors related to exoskeleton formation and the major transcriptional events during premolt, and their timing was determined [25]. Nevertheless, molecular molting mechanisms have been studied in other crustaceans and several molting-related factors have been characterized [26,27,28,29]. For example, Gao et al. (2015) identified several genes related to immunity, hormonal regulation, and other factors related to molting when they conducted a whole transcriptome analysis of the molting process [27]. Kuballa et al. identified factors related to energy cellular energy requirements cycle, cuticular protein, hemocyanin, cuticle hardening, muscle formation, and lipid metabolism across the different stages of the molting process [26].

To understand the mechanisms and molecular events related to crayfish molting, RNA sequencing (RNA-seq) was used to explore the expression changes of genes that occur between the intermolt stage and postmolt stage. Tissues from the hepatopancreas of the crayfish in the two molting stages were used for transcriptome sequencing. This work will contribute to a better understanding of the crustacean molting regulatory mechanisms, thereby shedding light on how molecular mechanisms can be used to intervene in the molting process for improved culture and management of red swamp crayfish and other crustaceans. Furthermore, our work provides a valuable resource of crayfish transcriptome genome annotation for further identification of candidate genes controlling molting in crayfish and other molting animals.

## 2. Materials and Methods

### 2.1. Experimental Animals

In total, 40 red swamp crayfish of the same developmental stage, weighing 10–25 g, were obtained from the greenhouse to the experimental laboratory at Freshwater Research Center of the Chinese Academy of Fishery Science (FFRC). The red swamp crayfish were put in four equal-size glass tanks at a density of 10 crayfish per tank and acclimated to the laboratory conditions for two weeks. After two weeks, the molting process was observed 24 h daily for three weeks, and all the crayfish were fed with the same commercial feed to satiation (three times a day; 9 a.m., 3 p.m., and 7 p.m.). The crayfish were cared for following the international and China’s guide for the care and use of experimental animals.

### 2.2. Sample Collection and Preparation

For this, 15 min after the molting process was observed (postmolt stage, designated as M), the red swamp crayfish were sampled and dissected to collect the hepatopancreas. To characterize the intermolt stage (designated as NM) of crayfish, the previously molted crayfish were put in a separate within the same tank and observed until a hard exoskeleton was formed, with no evidence of epidermal retraction [30], and they were sampled at the same time when the molted crayfish were sampled. The hepatopancreas tissues collected from crayfish in the two molt stages (post molt and intermolt stage) were stored in a −80 degrees freezer awaiting RNA extraction. A total of six crayfish were sampled, three (M1, M2, and M3) in the postmolt stage and the other three (NM1, NM2, and NM3) in the intermolt stage.

### 2.3. RNA Isolation and RNA-Seq Library Preparation

Approximately 80 mg of hepatopancreas tissue was used for RNA extracted using Trizol (Invitrogen, Carlsbad, CA, USA) following the manufacturer’s instructions. The quality and amount of RNA integrity were verified using an Agilent 2100 Bioanalyzer (Agilent, Shanghai, China). Following the manufacturer’s instructions, 3 μg RNA per group was used for sequencing libraries, which were generated using a NEBNext Ultra RNA Library Prep Kit for Illumina (San Diego, CA, USA) (New England Biolabs (NED), Boston, MA, USA). The index-coded samples were clustered on a cBot Cluster Generation System using the TruSeq PE Cluster Kit v3-cBot-HS (Illumina) as outlined by the manufacturer. Then, on an Illumina Hiseq × 10 platform, the library preparations were sequenced and 150 bp pair-end reads were obtained.

### 2.4. Transcriptome Assembly and Annotation

Raw reads from the two stages were combined and quality-filtered using the FastQC v0.11.2 and Trimmomatic read trimming tool (2.0–10) [31]. Using sliding window analysis, the 3′ and 5′ reads with an average quality score below 20 were trimmed, and reads with quality less than 10 at the beginning or the end were removed. The transcripts with a length of at least 100 bp were used for further analysis. The clean reads were used for the reference transcriptome assembly based on Trinity software (version 2.4.0), with the paired-end mode [32,33]. Using Bowtie 2.3.2, raw sequenced reads were mapped to the assembled reference transcriptome to filter out the misassembled transcripts [34]. To estimate the abundance of transcripts, RSEM software (version 1.3.1) was used [35], the fragments per kilobase per transcript per million mapped reads (FPKM) value was calculated and filtered out the transcripts with FPKM < 1. The filtered transcripts were used as *Procambarus clarkii* reference transcriptome for downstream analysis. Furthermore, the raw reads were mapped to the assembled transcriptome using Bwa-0.7.9a to examine the accuracy of the assembled transcriptome [36]. The mapping statistics were calculated using SAMtools (version 1.5) [37].

Using NCBI BLAST+ (version 2.60), against NR, Swissprot, GO, KEGG, and KOG databases, assembled transcripts Unigenes were annotated, with a cutoff E-value lower than 1 × 10^−6^. NCBI BLAST+ findings were imported into the BLAST2GO program (3.2) [38], and then the EC numbers for the KEGG pathway and Gene Ontology (GO) terms were annotated [39].

### 2.5. Analysis of Simple-Sequence Repeats (SSR), SNP, InDells, and Genebody Coverage

SSR from transcripts were examined by MIST. SNP/InDel calling was performed by BCFtools with the parameters: the quality score is more than 20 and coverage depth is more than 8. The annotation of these SNPs is shown in dataSNP_ anno.csv (Table S1). Using BEDTools, we calculated the coverage ratio to see the percentage of genes that were completely detected and not detected in each sample in this sequencing.

### 2.6. Analysis of Differentially Expressed Genes (DEGs)

Analysis of differentially expressed genes was performed using the DESeq R package (1.26) (1.26) [40], which provided the statistical basis for determining the differential expression in the samples. All the genes with *q*-Values < 0.05 and |fold change| > 2, were considered as differentially expressed.

ClusterProfiler [41] was used to classify the overrepresented GO terms in the biological process (BP), molecular function (MF) groups, and cellular component (CC), as well as the KEGG pathway categories, to determine the functions and significantly enriched pathways of the DEGs. For these analyses, the hypergeometric distribution threshold was a *p*-value of <0.05. GO enrichment analysis of the DEGs was conducted using the GOseq R package based [42].

### 2.7. Molting Pathway Analysis

To identify the most important molt-related genes and factors, a module analysis was performed using Cytoscape (version 3.7.1) [43]. Modules that are significant were identified using clustering score using the node score cutoff criteria. The higher the clustering scores of the node, the more important the factor is considered in the molting process, and hence, a threshold score of >6 was chosen. The interaction pathways for these factors were plotted. The factors in the selected nodes were subjected to transcription factor (TF)–target factors regulatory network analysis to determine the potential factors important in the molting process.

### 2.8. qRT–PCR

To validate RNA-seq data and expression, we chose eight genes that were significantly enriched in KEGG pathway analysis, and the 18S rRNA gene (**AF436001**) was used as an internal reference gene. The primers for the selected genes were designed using Primer3 Input (v. 4.1.0), (https://primer3.ut.ee/, accessed on 10 September 2019) (Table S2). Approximately, 2 µg of total RNA per sample was used to generate the first-strand cDNA using a reverse transcription system (Takara, Dalian, China). Quantitative RT–PCR was performed using SYBR Premix Ex Taq (Takara, Dalian, China) on ABI 7500 system. The amplification program was performed at 95 °C for 2 min, followed by 95 °C for 15 s and 60 °C for 31 s (40 cycles). Three biological replicates were performed for each gene. The relative expression levels of genes were calculated using the 2–CT method [44].

## 3. Results

### 3.1. Ecdysteroid Changes in Crayfish Morphology

**Figure 1:**
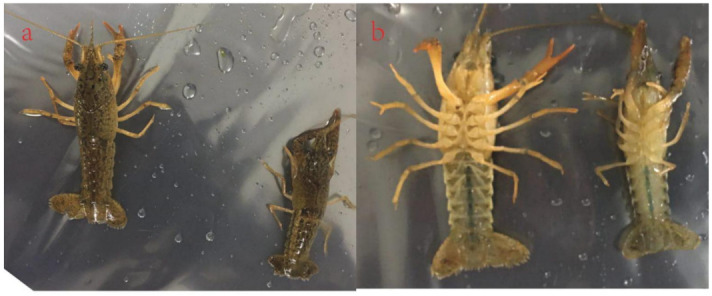
The morphological changes that occur during the molting process: (**a**) intermolt stage, in which the crayfish has a hard and calcified exoskeleton and (**b**) postmolt stage, in which the crayfish has just shed its exoskeleton, and its skin is soft.

In our study, we observed the molting process as it proceeded from the intermolt stage through to the postmolt stage. In the intermolt stage, the crayfish had a fully developed hard exoskeleton (Figure 1a), then the exoskeleton was partially digested during the premolt staging as it progressed toward molting. During the molt stage, water was absorbed to increase the body volume, leading to the splitting of the partially degraded old exoskeleton. After molting, the crayfish was very soft and vulnerable due to the exoskeleton, which was not calcified; it is at this stage that the exoskeleton and membranous layers begin to be formed (Figure 1b).

### 3.2. Read Sequencing, Assembly, and Mapping

For this study, the transcriptomes were obtained from the hepatopancreas of the crayfish in the postmolt and the intermolt. The acquired raw reads were trimmed and sifted using the Illumina sequencing platform, generating a total of 307,608,398 clean reads, with an average length of 145 bp (Table 1). The average GC content of the clean reads was 47.71%, while the GC% for intermolt (48.61%) was slightly higher than that of the postmolt (46.80%). The proportion of nucleotides with quality values larger than 30 in reads (Q30) was 95.06%, with the intermolt representing 94.60% and postmolt representing 95.51% (Table 1).

Using the Trinity program, a total of 272,346 transcripts were assembled from the clean reads, having an average length of 885 bp and an N50 of the length of 1806 bp. Of these transcripts, 41.5% were more than 500 bp in length and 24.3% longer than 1000 bp (Table 2). The transcriptomes of the two molting stages were de novo assembled in 139,100 unigenes.

These unigenes (139,100) had an average length of 676 bp, of which 33.1% were longer than 500 bp in length and 24.3% longer than 1000 bp (Table 2; Figure S1b). Furthermore, most of the identified genes had a length between 200 and 300, GC content within the range of 30–50%, and isoforms ranging between 2 and 3 (11.4%) (Figure S1d). Gene body coverage analysis was used to evaluate the two molting stages to see the skewness in the dataset to avoid biases and large variation. The results separated the samples into two classes; samples from the same molting stage showed a similar distribution pattern, and the curves clustered together (Figure S2a).

The clean reads for the two libraries (postmolt and the intermolt) were aligned against the de novo assembled reference transcriptome, in which we found that 94.7% of the postmolt reads and 95.7% of the intermolt reads were mapped. Among the postmolt reads (94.7%), 76.0% were multimapped, 18.7% uniquely mapped, and 16.6% were mapped in proper pairs. Of the intermolt reads (95.7%) mapped, 77.3% were multimapped, 18.4% uniquely mapped, and 15.9% were mapped in proper pairs. Splice reads for both libraries were 0%, while nonsplice reads were 18.7% and 18.4% for postmolt and the intermolt, respectively (Table S3). The assessment of the unique gene mapping ratio and the genome mapping ratio met the requirements for follow-up analysis.

### 3.3. Functional Annotation

The assembled unigenes were searched against the NCBI, NR, KEGG, Swissprot, and KOG databases, using the BLAST+ (E-value, 10-6). The annotation results showed that out of the 139,100 assembled unigenes, 5064 unigenes had homologous sequences in all the four databases; 96 unigenes in the KEGG, Swissprot, and KOG; 625 in the NR, KEGG, and Swissprot; and 3961 in the NR, Swissprot, and KOG. Furthermore, we found out that 6511 unigenes only had homologous sequences in NR, 5 unigenes only in KEGG, 570 unigenes only in Swissprot, and 74 unigenes only in KOG. The total number of unigenes with homologous sequences in each database were 19,217 in NR, 5863 KEGG, 13,484 Swissprot, and 9509 in the KOG database. The annotation results are illustrated in the Venn diagram (Table S4 and Figure S2b).

### 3.4. Characterization of SSR, SNPs, and InDels

The simple sequence repeats (SSR) ranged from one to six. The number of SSRs with each repeat unit was found to vary, the most common SSRs were mono-nucleotide repeats, followed by di-nucleotide repeats and the hexanucleotide repeats were the least common (Figure S3a). The SNPs and InDels distributions within various genomic features were revealed by annotation of the transcripts from all samples to the crayfish reference genome. Generally, a similar distribution pattern in both SNPs and InDels was observed in the six samples (Figure S3b). The SNPs and InDels were highest in M2, followed by M1, and were least in NM1. The average number of the molt stage SNPs was higher than that of NM. The annotation of these SNPs is shown in dataSNP_anno.csv (Table S1). By combining the annotation of DEG with these SNPs, dataSNP_anno3deg.csv was produced, which consisted of 712 genes. These genes were mainly divided into four groups—those promoting growth, directly linked to the molting process, immune-related genes, and those involved in lipid metabolism.

### 3.5. Differentially Expressed Genes (DEGs) Analysis

**Figure 2:**
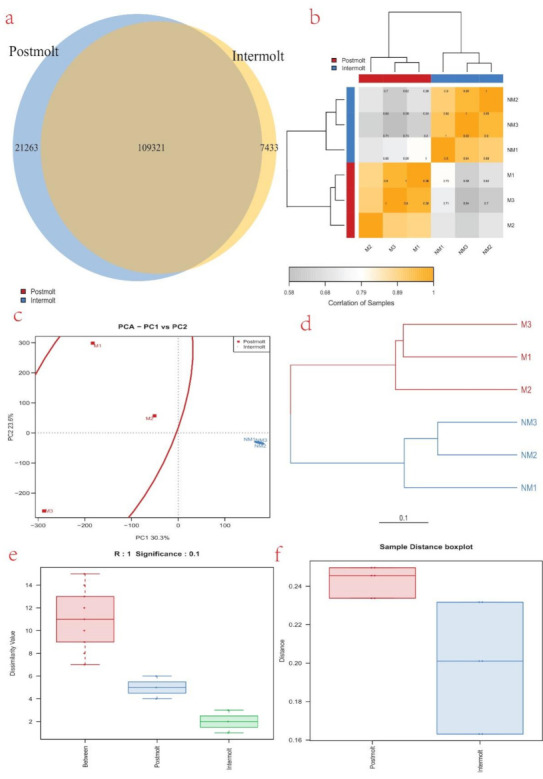
(**a**) Venn diagram showing the number of DEGs expressed in each molting stage (N vs. NM) and those expressed in both stages; (**b**) the DEGs correlation heatmap of sample clustering. The more similar the two samples are, the nearer the distance is. Each color box represents the distance between different samples. The greater the distance, the whiter, and the closer the distance, the deeper the yellow; (**c**) cluster analysis of the samples: PCA plot illustrates the similarities and differences of the samples from the two molting stags (M and NM); (**d**) cluster analysis of the samples: dendrogram is illustrating similarities and differences of the samples from the two molting stags; (**e**) dissimilarity box plot illustrating the relationship between samples from the two molting stages; (**f**) distance box plot illustrating the relationship between samples from the two molting stages.

Analysis of the two libraries showed that 130,584 genes were expressed in the postmolt stage (94.6% of the genes) and 116,754 were expressed in the intermolt stage (84.6% of the genes), among which 109,321 were expressed in both the postmolt and the intermolt stages. Furthermore, 21,263 were only expressed in the postmolt, while 7433 were only expressed in the intermolt (Figure 2a). Among the expressed genes, we found that 2347 were significantly differentially expressed, of which 1401 were upregulated and 946 were downregulated (Table S5). To understand and illustrate the relationship between the postmolt and the intermolt, we performed hierarchical clustering of the DEGs from all the samples, using Ward’s method of Euclidean distances (Ward, 1963) (Figure 2b). The results showed a higher correlation between samples in each molting stage; hence, big differences occur between the two molting stages. Similarly, the principal component analysis (PCA) plot revealed that the DEGs from all samples could be clustered into two groups, based on similarities of gene expression patterns (Figure 2c). In addition, a dendrogram placed the sample DEGs into two main clusters: M1, M2, and M3 in one cluster, and NM1, NM2, and NM3 in another cluster (Figure 2d). Similar trends were observed in the box plot of the sample DEGs (Figure 2e,f).

**Figure 3:**
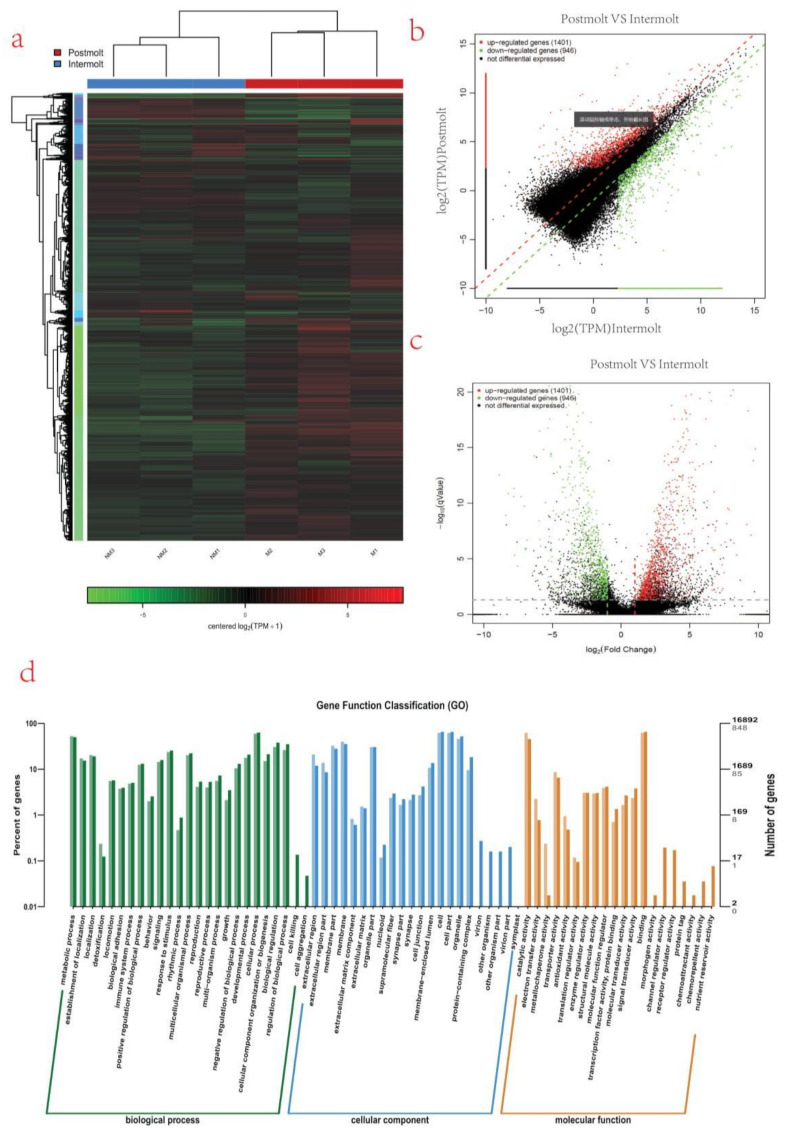
Differentially expressed genes and their GO annotation. (**a**) Heat map of differentially expressed genes, colored keys represent the fold changes (log2 transformed counts) of gene expression between the molt stages; red represents upregulation, and green represents downregulation. Each column represents the molting stage; (**b**) Scatter plot showing log2 (TPM) intermolt (x-axis) and log2 (TPM) molt (y-axis) of the differentially expressed genes, where red represent upregulation, green represent downregulation, and black represent nondifferentiated genes; (**c**) volcano plot showing log2 (fold change) (x-axis) and significance (−log10 * adjusted *p*-value; y-axis) of the differentially expressed genes, where red represent upregulation, green represent downregulation, and black represents nondifferentiated genes; (**d**) Gene Ontology (GO) classification of transcripts from the two samples (intermolt and postmolt). The three main GO categories include biological process (blue), cellular component (red), and molecular function (green).

A heat map that grouped genes according to FPKM values was generated in Cluster [45] and visualized in TreeView to analyze their expression levels across molting stages [46] (Figure 3a). It showed that the relative expression profiles of the transcripts in the two molting stages can be divided into two clusters, the postmolt and the intermolt stage. The significantly differentially expressed genes in the postmolt vs. intermolt are shown in the volcano plot (Table S5; Figure 3b,c). Of them, 261 differentially expressed genes were explored (Table S8 dat34.csv), and factors such as chitinase, lectin, cytochrome C, hemocyanin, etc., were found. The correlation ship between these differentially expressed genes were performed by Person’s correlation analysis (see Table S7 datDrp2r.csv). The correlated genes responded to molting related factors (hyperglycemic hormone, cryptocyanin 1 and hemocyanin) were selected and plotted (Figure S4). Several genes related to energy requirements, hormonal regulation, immune response, and exoskeleton formation were identified.

### 3.6. GO and KEGG Analysis of Differentially Expressed Genes

To acquire complete functional information and classification of postmolt vs. intermolt, unigenes were aligned against GO and KEGG databases. Gene Ontology (GO) analysis revealed that 16,892 unigenes were annotated. These unigenes were sorted into 67 functional groups that belong to GO categories including biological process, cellular process, and molecular function. In the cellular component category, the largest cluster of DEGs was associated with cell and cell parts. Under the biological process category, abundant DEGs were involved in the cellular process, development process, and biological process. Within the molecular function category, a large proportion was associated with catalytic activities and binding (Figure 3d).

**Figure 4:**
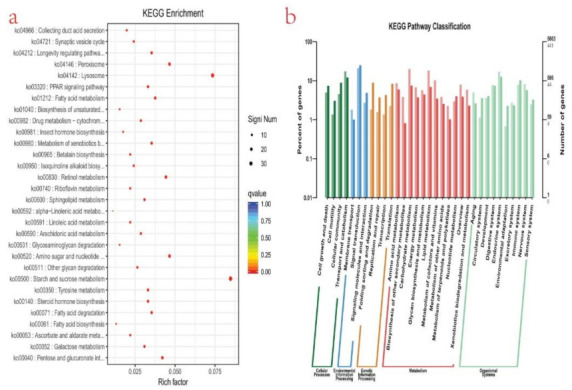
(**a**) Scatter plot of enriched KEGG pathways statistics. The rich factor is the ratio of the differentially expressed gene number to the total gene number in a certain pathway. Q-value is corrected *p*-value ranging from 0 to 1. The color and size of the dots represent the range of the Q-value and the number of DEGs mapped to the indicated pathways, respectively. Top 30 enriched pathways are shown in the figure; (**b**) plots showing categories of genes classified based on the Kyoto Encyclopedia of Genes and Genomes (KEGG) analysis; five categories were identified that significantly enriched function-to-function interaction network: The circular nodes are function information, and the edges represent the correlation between functions. The color of the node represents the degree of enrichment, i.e., the redder the color is, the higher the degree of enrichment, and the more yellow the color, the less the lower the degree of enrichment.

To understand the biological pathways of the genes further, it is necessary to carry out a pathway-based analysis (KEGG) [47]. For this purpose, 5863 unigenes were mapped to the reference of typical pathways in the KEGG database (Figure 4b). KEGG analysis revealed that 5863 unigenes were annotated. The DEGs were mapped to 33 different pathways, belonging to five KEGG pathways: cellular process, environmental processing information, genetic processing information, metabolism, and organismal system. To understand the biological processes related to the identified pathways of various DEGs further, KEGG enrichment analysis was conducted. A total of 30 KEGG processes were significantly enriched. The top 10 KEGG pathways of DEGs were enriched in starch and sucrose metabolism (ko00500), lysosome (ko04142), peroxisome (ko04146), retinol metabolism (ko00830), amino sugar and nucleotide (ko00520), *Pentose and glucuronate* interconversions (*ko00040*)*,* longevity regulating pathway (ko04212), metabolism of xenobiotics by cytochrome P45 (ko00980), fatty acid degradation (ko00071), and tyrosine metabolism ko00350) (Figure 4a).

The generated postmolt vs. intermolt transcripts were further evaluated, and several factors related to hormonal regulation, energy requirements, immune response, and skeletal-related activities were identified. A molting pathway diagram was plotted, showing the interaction of some of the identified molting-related factors (Figure S4). Five energy-related factors including NADH dehydrogenase, cytochrome p450, thyroid hormone signaling pathway, creatine kinase, and arginine kinase were identified. The expressions of energy-related factors are detailed in Table 3, Tables S6 and S8. Among the identified energy-related factors, only cytochrome and thyroid hormone signaling pathway were downregulated in the postmolt stage. The substantial variation in the expression of these factors in the two molting stages could be related to the fluctuating energy demands across the molt cycle.

To reveal the expression fluctuations of the immune-related factors between the two molting stages, we searched and evaluated molting-related immune factors identified and characterized in other arthropods with more focus on crustaceans. Approximately, nine immune-related factors including innate immune response, lysosome, Toll-like receptors, caspase, a trypsin-like serine protease, peroxiredoxin, thioredoxin, glutaredoxin, and c-type lectin-like were identified (Table 4, Tables S6 and S8). Interestingly, the majority of these immune-related factors were upregulated in the postmolt stage, compared to the intermolt.

Hormones are important regulators of the red swamp crayfish molting cycle, and they are involved are in numerous processes such as osmoregulation, reproduction, glucose metabolism, etc. [16,48]. To understand the global changes in the hormone regulation system that occur as the molting process progresses, we enumerated several hormonal-related factors. As a result, we identified eight types of molting factors related to hormone regulation (Table 5, Tables S6 and S8). Three of these factors were upregulated, and three others were downregulated in the postmolt stage, compared to intermolt. Two genes related to MIH and crustacean hyperglycemic hormone (CHH) were identified.

Expression patterns of factors implicated in exoskeleton formation were investigated to identify those that could be possibly be involved in the molting process. By data mining of the annotated transcripts with reference to previous studies of exoskeleton formation-related factors such as C-type lectin-like, mannose-binding protein, *Eriocheir sinensis* chitin synthase gene, CDA like 2, CBM 14, and cryptocyanin 1 were identified (Table 6, Tables S6 and S8). The expression profile of majority factors showed a characteristic pattern of upregulation at the postmolt stage, compared to intermolt. However, cryptocyanin 1 was downregulated at the postmolt stage.

### 3.7. Analysis of Transcriptome Data by qRT–PCR

**Figure 5:**
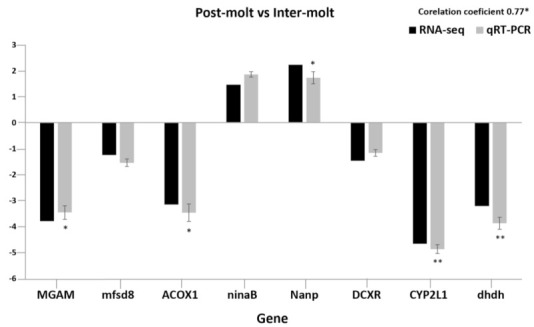
The expression patterns of the eight selected genes detected by the qRT–PCR and RNA-seq. Comparison of relative fold changes between qRT–PCR and RNA-seq results between the postmolt and intermolt stage. Fold changes are presented as the ratio of gene expression in the postmolt stages to the intermolt stage as normalized with the 18S rRNA gene (AF436001). The qRT–PCR data are mean + SEM. RNA-seq is transcriptome data, and qRT–PCR data are the expression patterns detected by the qRT–PCR method. Statistical significance of the qRT–PCR relative expression ratio is shown as (* *p* < 0.05; ** *p* < 0.01).

To validate the RNA-seq findings, we chose eight genes that were significantly enriched in KEGG analysis of the M vs. NM molting stages and performed RT–PCR. The selected genes include *MGAM*, *mfsd8, ACOX1, ninaB, Nanp, DCXR, CYP2L1, dhdh*, and *AF436001* (internal reference gene). The relative expression level for the selected genes was subjected to the Student’s *t*-test (*p* < 0.05, *p* < 0.01) to evaluate the statistical significance of the fold changes observed by qRT–PCR. The fold changes for all the genes measured by qRT–PCR were statistically significant (*p* < 0.05; *p* < 0.01, except for mfsd8, ninaB, and DCXR. The transcript levels determined by RNA-Seq and qRT–PCR were similar. The two methods had a significant correlation, with average coefficients of 0.77 for the selected gene. The quantitative PCR results for all the selected genes prove that the de novo transcriptome assembly and expression levels calculated were accurate. These results, therefore, exhibit that gene expression profiles in each organism are accurately reflected by the expression patterns of genes in the transcriptome (Figure 5).

## 4. Discussion

The value of understanding the molecular mechanisms underlying the molting process in crayfish and other decapods is appreciated for a variety of reasons including disease control, growth enhancement, exoskeleton formation, predation studies, etc. [48,49,50,51,52,53]. Nevertheless, the molecular mechanisms underlying the crayfish molting process remains poorly understood. Therefore, the present study was conducted to provide more insights into this area.

The molting process is divided into four continuous but distinct stages (intermolt, premolt, molt, and postmolt) [5,17]. In this study, analysis of the obtained transcripts, separated the samples into two groups, according to the molting stage of the sampled crayfish (Figure 2). A higher correlation between samples from each molting stage was observed. In the PCA plot, the proximity of the samples in each group shows the similarity of gene expression among them (Figure 2c). In the intermolt stage, the samples (NM1, NM 2, and NM 3) were widely scattered, showing that they were at different phases of progression of that molting stage; on the other hand, samples in the postmolt stage (M1, M 2, and M 3) were close to each other, indicating that the phase of progression was similar among them. A comparison of the gene expression profiles of the two molting stages showed that the postmolt stage had highly differentially expressed genes, compared to the intermolt stage (Figure 2b and Figure 3a). This could be attributed to the fact that the postmolt is a critical stage that may have more developmental and immune response genes involved [10,54,55].

### 4.1. Energy Demand during the Molting Process

Factors related to mitochondrial proteins, such as cytochrome and NADH dehydrogenase were identified (Table 3, Tables S4, S6, and S8). NADH dehydrogenase and cytochrome are two of the three energy-transducing enzymes in the mitochondrial electron transport chain [56,57,58]. NADH dehydrogenase was upregulated in the postmolt stage, compared to the intermolt stage. The expression profile of NADH dehydrogenase seems to reflect an increase in the energy requirements of the animal in the postmolt stage. This is because, in the postmolt stage, the animal is recovering from molting, and hence, a lot of metabolic activities are taking place, requiring a substantial amount of energy [10,55]. Interestingly, several studies have reported lower levels of energy requirements during molting, followed by a sharp rise in the postmolt stage due to recovery in the metabolic activities before returning to normal in the intermolt stages [10,26]. Cytochrome was downregulated in the postmolt stage when compared to the intermolt stage. In animals, cytochrome and NADH dehydrogenase are reported to be stimulated by thyroid hormones through the stimulation of mitochondrial activity [59]. However, in this study, the thyroid hormone signaling pathway was downregulated in the postmolt stage when compared to the intermolt stage. (ko04919; thyroid hormone signaling pathway).

Phosphagen kinases such as creatine kinase and arginine kinase were identified and found to be significantly upregulated in the postmolt stage, compared to the intermolt stage (Table 3). Phosphagen kinases serve in intracellular energy transport and as a temporal ATP buffer [60,61,62]. Phosphagen kinases are found in different parts of crustaceans; however, they are more abundant in muscles and gills [62]. Arginine kinase activity is found to vary significantly based on the tissue in *Carcinus maenas*, in which its abundance corresponds with the energy requirements of the tissue [63]. Therefore, given the fluctuation in energy demands during the molt cycle, we assume that phosphagen kinases play a key role as ATP buffer to meet the high energy demand in the postmolt stage.

### 4.2. Immunoregulation Process during Molting

Crustaceans have a lot of defense mechanisms that become activated depending on the stage of the molting process or the pathogen’s characteristics [64,65,66]. The defense mechanisms of crustaceans depend on the innate immune system, activated when pathogen-related molecular patterns are recognized by soluble or by cell surface proteins of the host, lectins, antimicrobial, etc.; these, in turn, activate humoral or cellular effector mechanisms to destroy invasive pathogens [66]. For this study, of particular importance was to learn the crayfish immune mechanism during the intermolt stage and the postmolt period. In crustaceans, several researchers have found that the molt and postmolt stages as periods when the animal is more susceptible to pathogenic infections and stress [67]. This is because its new exoskeleton is not well developed, a lot of physiological processes are taking place, and it has a low exercise capacity. In the present study, several immune-related factors were identified and were found to be highly upregulated postmolt, compared to the intermolt stage (Table 4, Tables S4–S6, and S8).

The GO term representing (GO:0045087), the innate immune response was highly expressed in the postmolt stage, representing an activated immunity to counter possible invasion during the stage. Based on the KEGG pathway analysis for DEGs between the postmolt and intermolt, some signaling pathways related to the innate immune system of the invertebrate were identified. Lysosome was one of the most significantly enriched KEGG pathways (Figure 4a). Activities related to lysozymes (GO:0003796), a product of lysosomes, were found to be highly expressed in the postmolt stage. The lysozymes are widely distributed immune effectors involved in numerous physiological processes, such as digestion and immune responses [68,69,70], exerting cytosolic activity on peptidoglycans of bacterial cell walls to initiate cell lysis. Moderate lysozyme antimicrobial activity was revealed in mud crab *(Scylla paramamosain)* [71]. The Toll-like receptor was another signaling pathway found to be significantly upregulated in the postmolt stage, compared to the intermolt. Toll-like receptors (TLRs) are a group of molecules that play an essential role in the recognition of pathogen-related molecular patterns (PAMPs) and the initiation of innate immune responses to infectious substances [72,73,74].

Caspase, interleukin 1 beta converting enzyme, was found to be upregulated in the postmolt stage. Interleukin 1 beta enzyme is a cysteine protease that converts pro-IL-1β into active IL-1β, a pro-inflammatory cytokine that mediates many physiological and behavioral responses to inflammation [75,76]. A study conducted recently showed that the IL-1 family may play an important role in activating innate immune responses against pathogen infection in mud crab [76]. Interleukin 1 (IL-1) family of cytokines, such as IL-1β and IL-16 are secreted when the NOD-like receptors (NLRs) detect intracellular bacterial and viral infections and induce the inflammasome complex [77].

Serine proteases (SPs) are among the biggest enzyme families in the animal kingdom and play essential roles in immune responses [78]. In this study, trypsin-like serine protease was upregulated in the postmolt stage. In crustaceans, trypsin-like serine proteases have been isolated from the hepatopancreas of the Chinese shrimp (*Fenneropenaeus chinensis),* and the red claw crayfish (*Cherax quadricarinatus)* were observed to involved in the innate immune defense against pathogens [78,79]. Serine proteinase homologs were also obtained from mud crab (*S. paramamosain*), and it has been suggested that *Sp*-SPH protein could bind to several bacteria and play a key role in host defense against microbe invasion [80].

Just before molting, during molting, and after molting, there is an increase in the metabolic activities in crustaceans, leading to an additional burden on the antioxidant system, which, in turn, increases the risk of oxidative stress [81,82,83]. Normally, oxygen consumption rises, reaching a peak shortly before ecdysis, and then declines rapidly after molting to increase again to basal levels in the intermolt stage [84]. Several antioxidant enzymes are secreted during molting; these enzymes help in compensating for the oxidative stress caused by metabolism or invading microorganisms (immune response) [85]. Here, we found that redoxin enzyme family including peroxiredoxin, thioredoxin, and glutaredoxin proteins were significantly upregulated in the postmolt stage, compared to the intermolt stage. Thioredoxin, with a redox-active disulfide bridge, is essential for sustaining the balance of reactive oxygen species and has a vital role on the immune system [86,87]. Thioredoxin has been characterized in mud crab, and it is believed to be a potential biomarker gene for environmental stress evaluation in marine species [88]. The peroxiredoxins (Prxs) define a novel and evolutionarily conserved superfamily of peroxidases able to protect cells from oxidative damage by catalyzing the reduction of a wide range of cellular peroxides [89,90]. Six peroxiredoxins were recently characterized in mud crab [91]. They were also characterized in crayfish during a bacteria challenge and other crustaceans [92]. Superoxide dismutase was also significantly upregulated in the postmolt stage, compared to the intermolt period. Superoxide dismutase is an enzyme that helps break down potentially harmful oxygen molecules in cells [90,91].

C-type lectins (carbohydrate-binding proteins) were downregulated in the postmolt stage, compared to the intermolt stage. C-type lectins are involved in immune function through the lectin-complement pathway, where mannose-binding lectin identifies infectious substances and in turn activates the PO system [27,93].

### 4.3. Exoskeleton Calcification and Sclerotization-Related Activities

Hardening of the new exoskeleton is a complex process that involves calcification (mineralization) and sclerotization [94,95]. Two factors implicated in the control of calcification in diverse structural matrices of invertebrates, C-type lectin-like domain, and mannose-binding protein were found to be significantly upregulated in the postmolt, compared to the intermolt, stage (Table 6, Tables S4 and S6). C-type lectin-like domain and mannose-binding proteins are relevant to the molt cycle-related modification of cuticular glycoproteins [96]. Glycosylation of cuticular proteins is believed to be a controller of biomineralization of the exoskeleton of crustaceans [97,98]. It has been suggested that glycoproteins serve as premolt inhibitors of calcification in the exocuticle and that deglycosylation postmolt may eliminate this barrier to mineralization [98]. C-type lectin-like is implicated in the inhibition of calcification by glycosylation of cuticular proteins since its expression coincided with the formation of the new cuticle in the premolt and must remain uncalcified until the postmolt [53]. The C-type lectin was found to be highly expressed in the premolt and dropped as the molt proceeded through to the postmolt stage and eventually reached lower levels in the intermolt stage. Similarly, our results show higher expression of C-type lectin in the postmolt stage, compared to intermolt. Since we did not sample crayfish in the premolt stage, we cannot determine the expression pattern of the C-type lectin across the molt stages; nonetheless, its expression in the postmolt stage could be attributed to the inhibition of calcification of the membranous layer, arthrodial membranes, gills, and the gut, which remain uncalcified at all the times. The upregulation of C-type lectin in the postmolt stage can also be attributed to the fact it plays a role in immune responses, as described earlier.

The expression of the mannose-binding protein, on the other hand, has been attributed to the facilitation of calcification of the exoskeleton through deglycosylation of cuticular proteins [53,99]. Kuballa and Elizur found that upregulation of mannose-binding protein was specific only to the postmolt period, at the time when cuticle calcification takes place [53], strongly suggesting its involvement in the calcification process. In this study, expression of the mannose-binding protein was more expressed in the postmolt stage than the intermolt; hence, we postulate that mannose-binding protein is involved in the calcification of the exoskeleton.

To understand the activities related to the exoskeleton formation further, the expression pattern of other factors such as chitin synthase, CDA, CBM, and cryptocyanin were evaluated (Table 6). Chitin synthase (CHS) is an essential membrane protein in arthropods, responsible for the synthesis of chitin from UDP-N-acetylglucosamine and its transfer to the extracellular domain [100]. Thus far, two chitin synthases are recognized in insects [101,102], one of which is involved in cuticle construction in larvae and adult arthropod peritrophic membrane [101]. In our results, chitinase was upregulated in the postmolt stage when compared to the intermolt stage, indicating its potential involvement in the formation of the new exoskeleton. CDA is one of the exoskeleton-related proteins identified in this study, and it was found to be significantly upregulated in the postmolt stage. CDA converts chitin into chitosan by digesting chitin acetyl groups. Hence, it has an important role in exoskeleton formation. Tom et al. annotated two CDAs, one of which was prominently expressed [25]. Furthermore, cryptocyanin involved in the formation of the new exoskeleton in crustaceans was identified [103]. However, it was downregulated in the postmolt stage, compared to the intermolt stage. We also found that the chitin-binding peritrophin-A domain (CBM 14) was upregulated in the postmolt stage, compared to the intermolt stage. CBM 14 is found in chitin-binding proteins, chiefly as peritrophic matrix proteins of insects, and these proteins function as peritrophic membrane and are important in the structural formation in insects [104]. Upregulation of CBM 14 in the postmolt stage reveals its involvement in cuticle synthesis and hardening.

### 4.4. Hormone Regulation Process during Molting

The crayfish molting process is controlled by a complex interplay of hormones that work together or independently throughout the molting cycle [13]. The signals that come by neuropeptide hormones activate a series of physiological activities associated with molting, including losing the extracellular cuticle, escaping from the confines of the cuticle relatively rapidly, taking up water, expanding the new flexible exoskeleton, and then quickly hardening it for defense and locomotion [5]. In our results, several neuropeptide hormones and hormone-related genes were identified and analyzed (Table 5).

It is well recognized that CHH and MIH peptide families are key hormones controlling the molting process in crustaceans [105]. MIH, secreted by a neuro-secretory center known as the X-organ/sinus gland complex (XO–SG) in crustacean eyestalks, belongs to the CHH family. Ablation of XO–SG has been shown to reduce MIH secretion, which, in turn, enables ecdysteroid synthesis and enhanced molt frequency [13]. MIH was upregulated in the postmolt stage, compared to the intermolt stage (Table S5). This was also observed in the study conducted by Qiao et al.; in *V.* *nipponense,* MIH expression increased rapidly to the highest level in the postmolt and remained lowly expressed in the intermolt stage and reached its lowest levels in the premolt stage [106]. Similarly, Huang et al., in a study on the green mud crab *Scylla paramamosain*, reported that MIH increased significantly as the molting process progressed from postmolt stage to intermolt stage, then reduced sharply at premolt stage [107]. This may be because the animal has just undergone molting and may not need to undergo molting just as soon; hence, the hormone is secreted at high levels. Moreover, it marks the beginning of the new molt cycle. In this study, we observed that growth gonad inhibiting hormone (GIH) upregulated (Table 5), exhibiting a molting pattern similar to that of MIH; hence, we assumed that they may have common functions. Similarly, Qiao et al. reported that GIH and MIH were closely related and postulated that they have similar functions [106].

To understand further hormones downstream of the molting process of the red swamp crayfish pathway and clarify possible roles in this process, we enumerated several other hormonal-related transcriptional factors of the two molting stages. According to the results, a trend of upregulated transcript levels was observed in the majority of the identified factors during the postmolt stage, compared to the intermolt stage (Table 6). Ecdysteroid regulated-like protein, ecdysteroid kinase, and E75 nuclear receptor were reported previously to be associated with molting, and they have been found to be related to hormonal control during molting [108]. In this present study, ecdysteroid-regulated-like protein, ecdysteroid kinase, and E75 nuclear receptor were highly expressed in the postmolt, suggesting that they could be involved in hormonal regulation during this stage. The vitellogenin gene (VG) is believed to be is an ecdysteroid reactive gene; in *Cherax quadricarinatus*, synthesis of the vitellogenin gene has been revealed to be induced by 20E during the premolt [109]. In this study, we observed a downregulated trend of the vitellogenin gene in postmolt, compared to the intermolt. Neuroparsins were first recognized as antigonadotropic factors that delay vitellogenesis in insects [110,111]. The inhibitory effect of neuroparsin on vitellogenesis and oocyte maturation was confirmed by RNA interference in female *Schistocerca gregaria* [112]. In contrast, neuroparsins were observed to play a positive role in the maturation of oocytes by igniting vitellogenin production in the hepatopancreas [113]. In this study, two genes encoding neuroparsin were identified—one was upregulated, while the other was downregulated.

### 4.5. DEGs and SNPs Probe for Molting-Related Factors

Through a combined annotation of DEG with SNPs, dataSNP_anno3deg.csv was produced, consisting of 712 genes. These genes were divided into four parts: (1) those promoting growth, e.g., mannanase [114], insulin-like growth factor-binding protein [115], regucalcin [116]; (2) directly linked to the molting process, e.g., crustacean molting protein- cryptocyanine [117], hemocyanin 2, [117]; (3) immune-related genes, e.g., macrophage migration inhibitory factor MIF1 [118], interferon-related developmental regulator 1 [118], heat shock protein [119]; (4) lipid metabolism: fatty acid-binding protein 1 [120], long chain fatty-acid–CoA ligase 4 [121], fatty acid synthase [122]. By KEGG annotation, 224 pathways were found, where starch and sucrose metabolism, lysosome [123], and retinol metabolism [124] were the top three enriched pathways. The Top1 pathway is about energy metabolism, where the Top2–lysosome surface serves as a platform for the assembly of major signaling hubs such as mTORC1, AMPK, GSK3, and the inflammasome [123]. The Top3–retinol metabolism has important roles in the development of the nervous system and notochord and many other embryonic structures, as well as in the maintenance of epithelial surfaces, immune competence, and reproduction [123]. Combining the three pathways and Nr annotation genes revealed that the nervous system, energy metabolism, and immune system were playing a key role in the complex molting process of crayfish. A single nucleotide substitution in the third intron of insulin-like growth factor 2 (*IGF2*) is associated with increased muscle mass and reduced subcutaneous fat in domestic pigs. This mutation disrupts the binding of the ZBED6 transcription factor and leads to a threefold upregulation of *IGF2* expression in pig skeletal muscle [125]. Thus, examining the relationship between candidate SNPs and their effect on the function of responded genes is an important research direction that can address the molting synchronization issue, e.g., cryptocyanine, hemocyanin 2.

## 5. Conclusions

Tracing the expression patterns of genes during molting can help in explaining and deducing the molecular mechanisms involved in the molting process. The information presented here provides a view of the hepatopancreas transcripts available in the postmolt and intermolt stage of crayfish, with respect to energy requirements, hormonal regulation, immune response, and skeletal-related activities during the postmolt stage and the intermolt stage. In the present study, some genes and other factors were detected; however, we mainly focused on highly expressed factors and those described in other studies as relevant to energy requirements, hormonal regulation, immune response, and exoskeleton in arthropods with much focus on those found in crustaceans. The expression pattern of these genes in the two molting stages is evident that molting is a complex process encoded by a large number of gene families, involving many mechanisms and requiring a strict control system.

## Figures and Tables

**Table 1:** *Procambarus clarkii* transcriptome sequencing and assembly.

	NM2	NM3	NM1	M1	M3	M2
Total Reads Count (#)	52,358,598	50,324,500	43,018,686	46,972,334	46,798,082	68,136,198
Total Bases Count (bp)	7.61 × 10^9^	7.27 × 10^9^	6.23 × 10^9^	6.79 × 10^9^	6.81 × 10^9^	9.9 × 10^9^
Average Read Length (bp)	145.3	144.43	144.87	144.58	145.59	145.35
Q10 Bases Count (bp)	7.61 × 10^9^	7.27 × 10^9^	6.23 × 10^9^	6.79 × 10^9^	6.81 × 10^9^	9.9 × 10^9^
Q10 Bases Ratio (%)	100.00%	100.00%	100.00%	100.00%	100.00%	100.00%
Q20 Bases Count (bp)	7.49 × 10^9^	7.16 × 10^9^	6.14 × 10^9^	6.69 × 10^9^	6.75 × 10^9^	9.77 × 10^9^
Q20 Bases Ratio (%)	98.48%	98.48%	98.45%	98.48%	99.07%	98.64%
Q30 Bases Count (bp)	7.2 × 10^9^	6.88 × 10^9^	5.89 × 10^9^	6.43 × 10^9^	6.6 × 10^9^	9.41 × 10^9^
Q30 Bases Ratio (%)	94.63%	94.64%	94.54%	94.61%	96.88%	95.03%
N Bases Count (bp)	2331	2248	1972	2022	52894	5878
N Bases Ratio (%)	0.00%	0.00%	0.00%	0.00%	0.00%	0.00%
GC Bases Count (bp)	3.72 × 10^9^	3.59 × 10^9^	2.97 × 10^9^	3.17 × 10^9^	3.19 × 10^9^	4.65 × 10^9^
GC Bases Ratio (%)	48.86%	49.35%	47.62%	46.67%	46.83%	46.91%

**Table 2:** Transcriptome assembly information.

	No.	≥500 bp	≥1000 bp	N50	N90	Max Len	Min Len	Total Len	Average Len
Transcript	272,346	112,989	66,293	1806	307	20,532	201	2.41 × 10^8^	884.82
Unigene	139.100	46,048	21,701	1111	263	20,532	201	93,974,306	675.59

**Table 3:** Expression of energy molting-related factors.

Factor Name	Number of Genes
NADH dehydrogenase	3
Cytochrome P450	8
Thyroid hormone signaling pathway	16
Creatine kinase	1
Arginine kinase	1
C-type lectin-like domain	1

**Table 4:** Expression of immune-related factors.

Factor Name	Number of Genes
Innate immune response	3
Lysosome	1
Toll-like receptors	4
Caspace	3
Trypsin-like serine protease	3
Peroxiredoxin	1
Thioredoxin	3
Glutaredoxin	1
C-type lectin-like	6

**Table 5:** Expression of hormone-related molt factors.

Factor Name	Number of Genes
MIH	2
Crustacean hyperglycemic hormone (CHH)	2
Hyperglycemic hormone-like peptide 2 precursor	2
Neuroparsin	2
Ecdysteroid regulated-like protein	1
Ecdysteroid kinase	5
Vitellogenin	1
17-Beta-dehydrogenase 8-like isoform X1	3
E75 Nuclear receptor	1

**Table 6:** Expression of exoskeleton-related factors.

Factor Name	Number of Genes
C-type lectin-like	6
Manose-Binding Protein	1
Eriocheir sinensis chitin synthase gene	1
CDA like 2	1
CBM 14	3
Cryptocyanin 1	5

## Data Availability

The datasets presented in this study can be found at NCBI (https://www.ncbi.nlm.nih.gov/biosample?LinkName=bioproject_biosample_all&from_uid=592222 (PRJNA592222), accessed on 10 September 2019). Files used in this study are contained within the manuscript.

## Supplementary Materials

The following are available online at https://www.mdpi.com/article/10.3390/life11060480/s1, Figure S1: (a) Bar graph of unit size against SSR number per Mbp of M vs. NM. (b) Statistics of single nucleotide polymorphisms (SNPs) and insertion/deletions (indels) in the transcriptome (N vs. M). Figure S2: (a) Reads coverages of gene body percentile. (b)Venn diagram of annotation results against NCBI, NR, KEGG, Swissprot, and KOG. The number in each color block indicates the number of unigenes that are annotated by single or multiple databases. Figure S3: Length and GC-content of All-Unigene. (a) Bar chart depicting length distribution of All-Unigene identified in this study (length vs. ratio of unigenes). (b) Bar chart depicting length distribution Bar chart depicting length distribution (length vs. number unigenes). (c) GC content frequency distribution of All-Unigene of this study. (d) Isoform distribution of the unigenes. Figure S4: Putative molting genes related pathway, red edge represent negative correlation coefficient, green edge represent positive correlation coefficient. Table S1: Crayfish dataSNP anno.csv, Table S2: Primers used in the study. Table S3: Mapping statistics, Table S4: Annotation summary, Table. S5: Significantly differentiated genes post-molt vs. inter-molt analysis, Table S6: Energy, immune, hormonal regulation, and exoskeleton related factors, Table S7: Significantly correlation pairs among the differential expressed genes had crustacean annotation, Table S8: Differential expressed genes were listed according to the crustacean annotation, Video S1: Crayfish Molting process.
